# Alteration of viral lipid composition by expression of the phospholipid floppase ABCB4 reduces HIV vector infectivity

**DOI:** 10.1186/1742-4690-5-14

**Published:** 2008-02-01

**Authors:** Niek P van Til, Kirstin M Heutinck, Roos van der Rijt, Coen C Paulusma, Michel van Wijland, David M Markusic, Ronald PJ Oude Elferink, Jurgen Seppen

**Affiliations:** 1 AMC Liver Center, Meibergdreef 69, 1105 BK. Amsterdam, the Netherlands

## Abstract

**Background:**

The presence of cholesterol in the Human Immunodeficiency Virus (HIV) lipid envelop is important for viral function as cholesterol depleted viral particles show reduced infectivity. However, it is less well established whether other viral membrane lipids are also important for HIV infection.

The ABCB4 protein is a phosphatidyl choline (PC) floppase that mediates transport of PC from the inner to the outer membrane leaflet. This property enabled us to modulate the lipid composition of HIV vectors and study the effects on membrane composition and infection efficiency.

**Results:**

Virus generated in the presence of ABCB4 was enriched in PC and cholesterol but contained less sphingomyelin (SM). Viral titers were reduced 5.9 fold. These effects were not observed with an inactive ABCB4 mutant. The presence of the ABC transport inhibitor verapamil abolished the effect of ABCB4 expression on viral titers.

The ABCB4 mediated reduction in infectivity was caused by changes in the viral particles and not by components co purified with the virus because virus made in the presence of ABCB4 did not inhibit virus made without ABCB4 in a competition assay.

Incorporation of the envelope protein was not affected by the expression of ABCB4. The inhibitory effect of ABCB4 was independent of the viral envelope as the effect was observed with two different envelope proteins.

**Conclusion:**

Our data indicate that increasing the PC content of HIV particles reduces infectivity.

## Background

Because HIV budding takes place at specialized membrane microdomains which are enriched in cholesterol and sphingolipids (rafts), the lipid content of HIV reflects the composition of these membrane domains [1-4]. However, accumulating evidence suggest that retroviral membrane composition is not just a reflection of the producer cells' membrane but that components of the viral membrane play an important part in the viral life cycle.

HIV membrane cholesterol has been shown to be important for viral integrity and function, depletion of cholesterol from HIV by incubation with cyclodextrin results in altered morphology and reduced infectivity of the viral particles [2]. The presence of phosphatidylserine (PS) in the viral membrane is essential for infection of monocytes, but not T cells, by HIV[5]. Whether other membrane components of HIV are also important is less well established.

The importance of the lipid composition of target cells is somewhat better understood. Treatment of target cells with sphingomyelinase and cholesterol oxidase reduces the susceptibility of these cells to HIV by changing the conformation of HIV chemokine co-receptors [6]. Target cells with artificially increased levels of ceramide are less well infected by HIV because they endocytose viral particles more efficiently[7].

Phosphatidylserine is also important for infectivity, incubation of target cells with liposomes composed of PS increased their susceptibility to transduction with a variety of retroviral vectors[8]. In HIV, PS present in the viral membrane has been shown to be important for the infection of monocytes[5].

Together, these studies indicate the importance of HIV and target cell membrane composition for viral function and suggest that a better understanding of the role of different lipids in the HIV life cycle might lead to the development of novel therapeutics to combat HIV infection.

The ABCB4 membrane transporter, formerly designated as multidrug resistance protein 3 or Mdr3, mediates the outward translocation of phosphatidylcholine (PC) across the canalicular membrane of the hepatocyte [9]. This process is called lipid flopping, as opposed to translocation from the outer to the inner leaflet which is called flipping. In the liver, the translocation of PC from the hepatocytes into the bile is necessary to neutralize the toxic detergent action of bile salts. Lack of ABCB4 expression leads to the severe liver disease Progressive Familial Intrahepatic Cholestasis type 3 (PFIC3) [9].

In our efforts to develop gene therapy for PFIC3 we were unable to generate HIV ABCB4 vectors at sufficient titers. Because ABCB4 was expressed during vector production we investigated whether ABCB4 caused changes in the viral lipid composition and whether these changes were responsible for the reduced viral infectivity.

## Results

### ABC transport protein expression

Our efforts to develop lentiviral gene therapy for PFIC3 were unsuccesful because we were unable to produce ABCB4 expression vectors at sufficiently high titers. Because ABCB4 acts as a phosphatidylcholine floppase we hypothesised that the low viral titers were caused by ABCB4 mediated changes in the phospholipid composition of the lentiviral membrane. We therefore expressed wild type and mutant ABCB4 during viral vector production. The inactive ABCB4 mutant was constructed by substituting the essential lysine in the Walker A motif by methionine [10]. As an additional control we also expressed the related ABC transporter ABCC1 during viral vector production.

**Figure 1:**
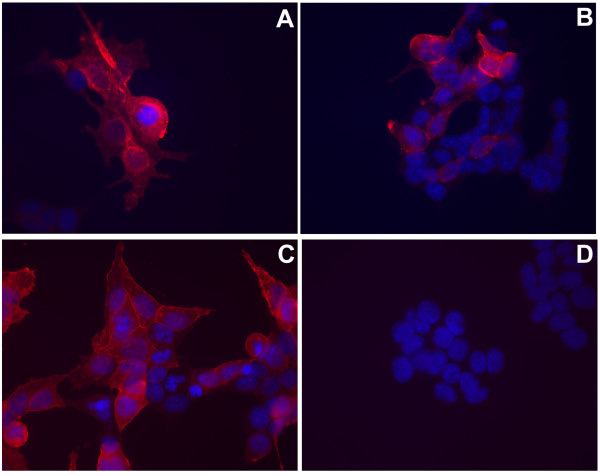
**Subcellular localisation of ABC transporters**. Cells transfected with ABCB4 or ABCC1 expression vectors were stained using antibodies to ABCB4 or ABCC1. (A) Wild type ABCB4, (B) mutant ABCB4. (C) ABCC1, (D) untransfected 293T cells. Nuclei were stained with DAPI. Strong immunoreactive staining was observed in cells expressing ABCB4, ABCB4mut and ABCC1.

We expressed wild type and mutant ABCB4 and wild type ABCC1, in 293T cells by transient transfection. Transfected cells were analysed by immunofluorescent staining for ABCB4 and ABCC1. Figure 1 shows that no gross differences in subcellular localisation were observed between the different proteins.

**Figure 2:**
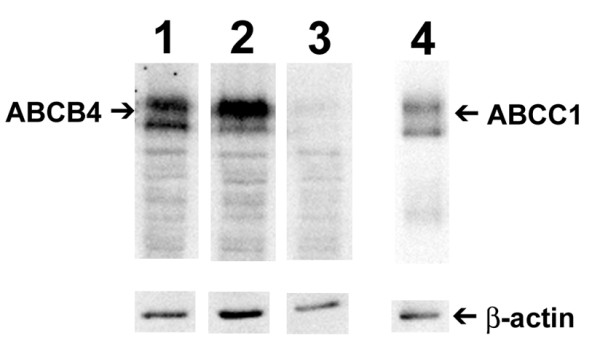
**Western blotting of ABC transport proteins in virus producing cells**. Lysates of 293T cells producing lentiviralvectors with or without co-transfection of ABC transporters were subjected to western blotting as described. Lanes represent: (1) wild type ABCB4, (2) mutant ABCB4, (3) negative control and (4) ABCC1. Wild type and mutant ABCB4 migrated at an apparent molecular mass of 140 kDa. ABCC1 migrated at a molecular mass of 170 kD. The two bands in both ABCB4 and ABCC1 lanes most likely correspond to differently glycosylated forms. Wild type and mutant ABCB4 appear to be differently processed. Similar amounts of cellular protein were loaded as shown by β-actin antibody reactivity.

Expression of ABC proteins was also analysed by Western blotting. Figure 2 reveals comparable expression levels of mutant and wild type ABCB4. Two bands are observed, likely representing variable glycosylation of the protein. Because the relative intensity of these bands is not the same, it seems that mutant and wild type ABCB4 are processed differently.

### Effect of ABC transport protein expression on viral titers

Next we examined the effect of wild type and mutant ABCB4 and ABCC1 expression on viral vector production. Viral titers and p24 output were determined. Table 1 shows that while the output of p24 is not affected by co expression of any of the ABC transport proteins, the viral titers are significantly reduced in the presence of wild type ABCB4. Because mutant ABCB4 is expressed at equal levels and at similar subcellular localisation as wild type ABCB4, the presence of an additional ABC transport protein in the transfected cells is not responsible for the observed reduction in viral titers. These experiments therefore indicate that co expression of ABCB4 results in less infectious viral particles.

**Table 1:** Co-expression of wild type ABCB4 reduces viral infectivity.

	TU/ml^a^	p24/ml^a^	TU/pg p24
Empty vector (n = 6)	100 %	100 %	9.9 ± 2.8**
ABCB4 wild type (n = 8)	17.0 ± 9.0 %	90.5 ± 55.3 %	2.3 ± 0.7
ABCB4 mutant (n = 8)	88.9 ± 39.8 %*	104.6 ± 30.3 %	8.9 ± 2.8**
ABCC1 (n = 7)	106.1 ± 48.4 %**	119.3 ± 66.9 %	7.8 ± 1.1**

PGKGFP virus was produced with wild type ABCB4, mutant ABCB4 or with ABCC1. The amount of p24 per ml and the viral titers were determined. Co expression of ABCB4 during viral production significantly reduces viral vector infectivity.

^a^The data for TU/ml and p24/ml are presented as a percentage, with the co-transfection of empty vector during the production of lentiviral vectors given an arbitrary value of 100%. Significant difference p < 0.01* or p < 0.001** compared to ABCB4 co-expression. Mean values are presented ± SD.

### ABCB4 co expression also reduces titers of virus pseudotyped with gp64

To show that the ABCB4 mediated reduction of viral infectivity is independent of the viral envelope, we generated GP64 pseudotyped virus in the presence of wild type and mutant ABCB4. GP64 pseudotyped virus produced with mutant ABCB4 had a titer of 5.1 ± 3 * 10^5 ^TU/ml whereas virus produced with wild type ABCB4 had a 22 fold reduced titer of 2.3 ± 3 * 10^4 ^TU/ml. Values are the averages of three independent experiments.

### The effect of ABCB4 expression on viral titers is the result of ABCB4 transport activity

To provide further evidence that ABCB4 activity is responsible for the reduction in viral vector titers we generated lentiviral vectors with wild type and mutant ABCB4, as described above, in the presence of the ABC inhibitor verapamil. Verapamil is a substrate and inhibitor of ABCB1 but also inhibits ABCB4 [10].

Table 2 shows that verapamil completely abolishes the effect of ABCB4 expression on viral infectivity. This result indicates that the transport activity of ABCB4 is responsible for the reduced viral titers.

**Table 2:** Verapamil inhibits the effect of ABCB4 expression on lentiviral vector infectivity.

Pseudotyping	ABCB4 mutant	ABCB4 wild type
VSVg (n = 2)	6.1 * 10^4^	8.2 * 10^4^
GP64 (n = 1)	9.1 * 10^4^	7.7 * 10^4^

Viral vectors pseudotyped with VSVg or GP64 were produced in the presence of wild type or mutant ABCB4 in the presence of Verapamil. Titers are expressed as HeLa transducing units/ml.

However, some caution with the interpretation of these results is needed. Typical titers of GP64 and VSVg pseudotyped virus are between 5 * 10^5 ^– 5 * 10^6 ^transducing units per ml, the titers reported in table 2 are at least tenfold lower. Thus, Verapamil abolished the effect of ABCB4 expression during virus production by lowering the titer of virus produced with mutant ABCB4 only. This reduction of titers is very likely caused by toxicity of Verapamil. Titers of virus produced with wild type ABCB4 were identical whether Verapamil was present or not.

### ABCB4 co expression during viral vector production does not impair viral envelope incorporation

**Figure 3:**
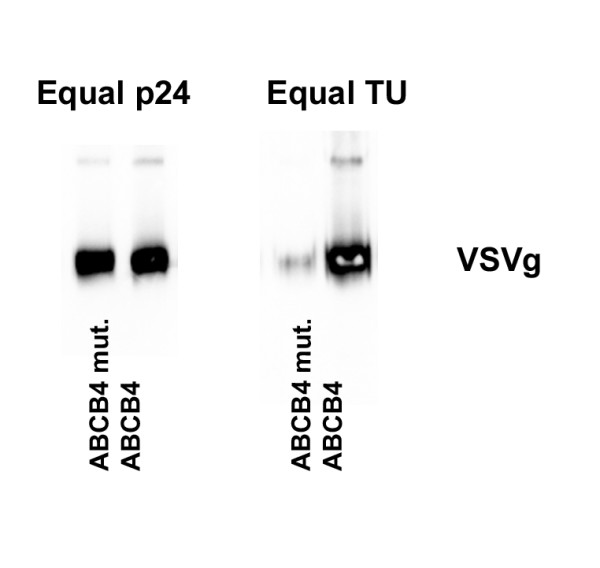
**VSVg incorporation in viral particles**. Virus produced with wild type and mutant ABCB4 was concentrated and VSVg protein was detected by Western blotting. When equal amounts of HIV p24 were loaded on the gel, the intensity of VSVg staining is also identical. With loading of equal amounts of transducing units (TU) the intensity of VSVg staining is much stronger in the virus produced with ABCB4. This indicates that ABCB4 expression during production of viral vectors does not compromise VSVg incorporation.

To exclude the possibility that the ABCB4 expression effected the incorporation of the VSVg envelope in the viral particles, we concentrated virus and analysed VSVg incorporation by Western blotting. Figure 3 shows that, when equal amounts of p24 are loaded, the same amount of VSVg protein is detected on Western blot. When equal amounts of transducing units are loaded, the amount of VSVg in the virus produced with wild type ABCB4 is much greater. This result demonstrates that ABCB4 expression does not impair viral envelope incorporation.

### PC lipososomes or viral supernatant generated in the presence of ABCB4 expression do not inhibit viral infectivity

Cells overexpressing ABCB4 might produce PC or PC vesicles that co purify with the viral vectors and inhibit infectivity. Thus, the reduced infectivity of virus produced with ABCB4 could be caused by changes in the viral particle or by the presence of these inhibiting membrane components produced by the viral producer cells.

To exclude the possibility that inhibiting components present in the viral preparations were responsible for the observed reduced infectivity we performed competition experiments.

Cells were transduced with GFP lentiviral vectors in the presence of PC liposomes or in the presence of viral supernatant generated with wild type or mutant ABCB4. The PC liposomes were added at a 1 μM concentration, similar to the amount measured in viral supernatants. Competing DsRed virus, produced with wild type or mutant ABCB4, was added at a saturating multiplicity of infection (MOI) of 5 for mutant and at identical amounts of p24 for wild type.

The control transductions, without addition of PC vesicles or with addition of CMVDsRed virus produced with mutant ABCB4, were given an arbitrary value of 100%. Transduction efficiency in the presence of PC liposomes was 94 ± 5 % and transduction efficiency in the presence of competing DsRed virus, produced with wild type ABCB4, was 96 ± 4 % of that of control virus. Values were derived from three independent experiments.

We therefore show that neither PC liposomes nor competing CMVDsRed viral supernatants significantly inhibited GFP vector transduction. These results also confirm earlier reports that show no effect of PC liposomes on lentiviral vector and HIV infectivity[8].

This experiment thus indicates that the reduced infectivity of virus produced with wild type ABCB4 is caused by changes in the viral particle and not by the presence of competing components in the viral preparations.

### ABCB4 overexpression changes lipid composition of lentiviral vectors

Next we determined whether ABCB4 overexpression during viral vector production mediated changes in the lipid composition of the viral particles.

Table 3 shows that the PC and cholesterol content of viral fractions is significantly increased by co expression of wild type ABCB4.

**Table 3:** Phosphatidylcholine and cholesterol content of lentviral vectors is increased by co expression of ABCB4 during production.

	nmol PC/μg p24	nmol cholesterol/μg p24
ABCB4 wild type (n = 6)	51 ± 26	40 ± 19
ABCB4mutant (n = 6)	4 ± 3*	9 ± 2*
Vector (n = 3)	6 ± 4*	8 ± 1*

PC and cholesterol content of lentiviral vector preparations produced with co-expression of ABCB4, ABCB4mutant or no co-expression. The values were adjusted for the amount of p24-gag antigen per sample. *Significant difference p < 0.01 compared to ABCB4 co-expression. Mean values are presented ± SD.

**Figure 4:**
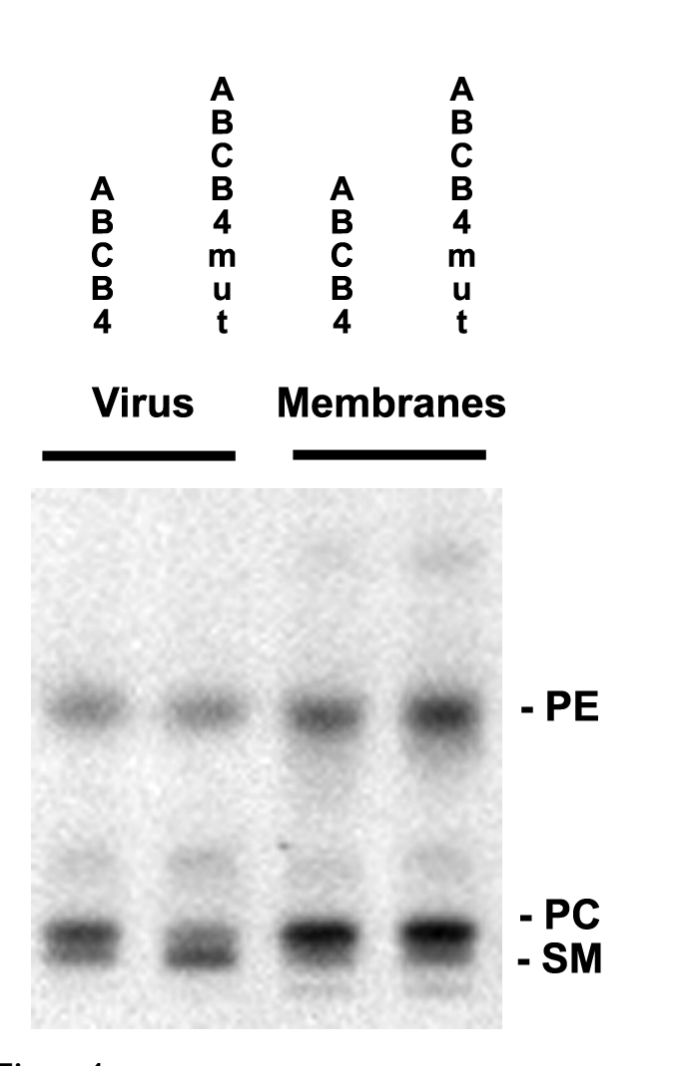
**Thin layer chromatography (TLC) of viral and cellular membranes**. Lipids from virus produced with wild type and mutant ABCB4 and membranes from the cells producing these viruses were isolated and analysed by TLC. Equal amounts of PC were loaded. The position of the different lipids is indicated. PE: phosphatidyl ethanolamine. PC: phosphatidylcholine. SM: sphingomyelin. Virus produced with wild type ABCB4 contains less SM.

To further analyse the lipid content we also fractionated the viral lipids by Thin Layer Chromatography (TLC) and compared these lipid profiles to that of the producer cells from which they were generated. Figure 4 shows lipid analysis by TLC of viral and cellular membranes. As expected, the viral fractions are enriched in sphingomyelin (SM) as compared to the cell membranes. Virus produced in the presence of wild type ABCB4 contains significantly less SM.

In table 4 the ratio's of the main membrane lipid constituents are depicted.

**Table 4:** ABCB4 co expression increases the PC/SM ratio of lentiviral vector particles

	ABCB4 mutant, virus (n = 3)	ABCB4 wild type, virus (n = 3)	ABCB4 mutant, 293T (n = 2)	ABCB4 wild type, 293T (n = 2)
PC/PE	1.3 ± 0.3	1.8 ± 0.4	1.8 ± 0.1	1.6 ± 0.1
PC/SM	2.1 ± 0.6	4.1 ± 0.7*	6.5 ± 4	7.8 ± 5

Lipids were extracted from virus and from membranes of virus producing cells. The ratio's of phosphatidylcholine (PC) phosphatidyl ethanolamine (PE) en sphingomyelin (SM) were determined by TLC as described. * p < 0.05 vs ABCB4 mutant virus.

A significant difference in the PC/SM ratio of virus produced in the presence of wild type and mutant ABCB4 is observed. This was expected since the virus produced with wild type ABCB4 contained a greater amount of PC. However, the ratio of PC to phosphatidylethanolamine (PE) is similar in viral and cellular membranes. These results are in agreement with a previous report on which lipid content of purified HIV and membranes from cells producing HIV. This study also showed that viral membranes were enriched in cholesterol and SM but that the ratio of PC to PE was identical in cellular and viral membranes[3].

Together our data indicate that expression of wild type ABCB4 during virus production mediates specific changes in the membrane composition of viral particles.

We conclude that the increased PC and cholesterol content and the decreased SM content of viral particles are responsible for the reduced infectivity.

## Discussion

Our results provide further evidence that the membrane composition of HIV is critical for infectivity. By co expression of ABCB4 during viral production we were able to change the membrane composition of lentiviral vector particles: The PC and cholesterol content was increased and the SM content was decreased. The result of this change was a strong decrease in viral infectivity. Because ABCB4 expression did not reduce the output of viral p24 and because we showed that the reduced infectivity was not caused by inhibitory compounds present in media from ABCB4 expressing cells, we can conclude that the change in lipid composition renders viral particles less infectious. The effect of ABCB4 on viral titers was independent of the viral envelope used. Thus, the viral membrane composition is an important factor in viral infection, independent of viral receptors.

Our study was performed with VSVg pseudotyped HIV particles. There is evidence that VSVg pseudotyped HIV is a good model to study effects of lipid composition on HIV infectivity: Cholesterol depletion also decreased infectivity of VSVg pseudotyped HIV [11]. However, because the effect of cholesterol depletion on VSVg pseudotyped HIV was less pronounced than on wild type HIV, the relevance of our findings for HIV biology must be determined in further studies with wild type HIV.

The exact mechanism by which the changed membrane composition affects viral infection is currently unknown. Because envelope incorporation in the viral particles is not affected by ABCB4 co expression, it is unlikely that binding of the virus is changed. As the effect occurs with different envelope proteins, it is also independent of the viral receptor. The most likely aspect that is disturbed would therefore be the viral membrane integrity. Studies in model membranes that resemble natural membranes, show that a decrease in SM and an increase in cholesterol will destabilize membranes [12]. Thus, it might well be that the change in lipid composition that is mediated by overexpression of ABCB4 during viral production would lead to a "leaky" viral particle.

Membrane rafts have been detected in HIV particles and disruption of viral rafts has been shown to decrease viral infectivity [13]. Because sphingomyelin is an important raft component, it could well be that the ABCB4 mediated decrease of sphingomyelin content decreases infectivity by disruption of viral raft domains.

The effect of two related ABC transport proteins, ABCB1 and ABCC1, on HIV production was investigated in two studies. A paper by Lee et al. reported the surprising observation that ABCB1 and an inactive mutant of ABCB1 both inhibited HIV production [14]. We clearly show that ABCB4 activity is responsible for the reduction of viral infectivity: an inactive mutant ABCB4 has no effect and the ABC protein inhibitor verapamil abolishes the effect of ABCB4.

An increase in HIV production by expression of ABCC1 was reported by Speck et al [15] However, in our hands ABCC1 has no effect on HIV vector production.

The studies of Speck and Lee were performed with replication competent HIV which makes it difficult to distinguish between the effect of ABC protein expression on viral production and infection. In our system there is no viral replication and we could therefore investigate the viral infection process only. ABCB1 transport protein expression in target cells has been reported to change HIV receptor expression which would lead to reduced infection and may therefore explain the discrepancy between our results and those of Lee et al [16].

A separate line of evidence also indicates that expression of ABC transport proteins in target cells decreases infectivity; incubation of hematopoietic cells with the broad ABC transport protein inhibitor verapamil increased HIV vector infectivity[17].

Our results also shed some light on ABCB4 function. A previous report showed that expression of ABCB4 in cultured cells was not sufficient to drive PC secretion. The presence of a PC acceptor in the culture media was required to measure PC efflux in ABCB4 overexpressing cells[10,18]. Our result suggest that overexpression of ABCB4 in the presence of a driving force for membrane budding such as HIV p24 production results in PC enrichment of the HIV lipid envelope. Expression of ABCB4 also leads to enrichment of cholesterol in the viral membranes. Although cholesterol secretion in bile of ABCB4 knockout mouse is disturbed [9], it can be restored to normal levels by infusing bile salts. This shows that, in the presence of a cholesterol "acceptor" ABCB4 function is not required for cholesterol secretion. We therefore presume that the ABCB4 mediated cholesterol secretion to HIV membranes is the result of co transport with PC.

## Conclusion

The experiments described in this paper document that an increase in PC and cholesterol of the HIV membrane inhibits viral infectivity. This finding may be potentially useful in the development of new classes of anti HIV drugs.

## Methods

### Construction of expression vectors

The mammalian expression plasmid pcDNA3.1+ (Invitrogen) was used to express the ABC transporters ABCB4, mutant ABCB4 and ABCC1. For the preparation of pcDNA3.1+-ABCB4, human wildtype ABCB4 cDNA [19] was cloned as an AgeI and XbaI fragment into the mammalian expression plasmid pcDNA3.1+.

The human ABCC1 cDNA [20] was cloned as a BamHI and NotI fragment into pcDNA3.1+.

To obtain inactive mutant ABCB4, a PCR was performed with the following oligonucleotide bearing a mismatch base CT CGA GCT AAC GTC AAG ATC TTG AAG GGC CTC AAC CTG AAG GTG CAG AGT GGG CAG ACG GTG GCC CTG GTT GGA AGT AGT GGC TGT GGG ATG AGC ACA ACG G and CAC GTC CAA TGG CGA TCC TC, to substitute nucleotide 1303 from adenine to thymine in the conserved Walker A domain, leading to an amino acid change at position 435 of a lysine into a methionine (mutation is underlined). This mutation was shown to completely inactivate ABCB4 [10] and the homologous transporter, ABCB1, as has been described previously[21,22]. The PCR product was cloned into pcR-TOPO2.1 (Invitrogen) and the correct substitution was confirmed by sequence analysis. A 2271 bp fragment was removed from pcDNA3.1+-ABCB4 by ApaI digestion and cloned into the pcR-TOPO2.1 vector that contained the mutated PCR fragment. From this plasmid a 2640 bp fragment was taken out by XhoI and XbaI restriction enzymes and ligated in place in the pcDNA3.1+-ABCB4 plasmid resulting in the mutatant ABCB4 (ABCB4mut).

### Lentiviral vector production

Third generation lentiviral particles pseudotyped with Vesicular Stomatitis Virus G-glycoprotein (VSV-G), were produced by transient transfection of 293T cells, and titrated on HeLa cells, as described [23]. The transfer vector PGKGFP with the phosphoglycerate kinase promoter driving GFP expression, was used in all gene transfer experiments [23]. For the competition experiments a lentiviral transfer vector was used in which the CMV promoter was driving the red fluorescent DsRed protein, CMVDsRed.

To determine the effect of ABCB4 or ABCC1 on viral vectors, lentiviral supernatants were generated by cotransfection as described above with the addition of 20 μg of ABCB4 or ABCC1 expression vector in the transfection mix.

In some viral production experiments the ABC protein inhibitor Verapamil was used in a concentration of 50 μg/ml during and after transfection.

Lentiviral vectors pseudotyped with Autographica californica GP64 envelope were generated by cotransfection as described above but with equal amounts of GP64 expression vector substituted for VSVg expression vector.

Viral particles were concentrated for Western blot and lipid assays by centrifugation in a SW28 rotor at 20,000 r.p.m. for 2 h at 4°C. The supernatant was removed after concentration, and the viral pellet was resuspended in PBS containing 1% sodium dodecyl sulphate (SDS). Samples were stored at -80°C.

The HIV-1 p24 Elisa kit (Perkin Elmer) was used to determine the amount of p24 in the viral supernatants according to instructions provided by the manufacturer.

### Western blots

Transfected cells were harvested in 2% SDS, and an equal volume of lysis buffer (20 mM KCl, 3 mM MgCl_2_-6H_2_O, 20 mM Tris/HCl, pH 7.4) with protease inhibitor mix (Roche). After sonication, protein content was determined by bicinchoninic acid protein assay kit (Sigma). For cell lysates, equal amounts of protein were loaded. For concentrated virus, equal amounts of transducing units or p24 were loaded. The gels were run, blotted, probed and developed as described [24].

The following antibodies were used: mouse anti-ABCB4 (P3_II_26, 1:1000) [25], rat anti-human ABCC1 (R1, 1:1000)[26], Rabbit anti VSVg (Sigma) and mouse anti-β-actin (AB-5, 1:1000, Neomarkers).

### Immunofluorescence staining of transfected cells

The 293T cells were seeded 1 × 10^5 ^in 6-well plates on rat-tail collagen coated glass slides and transfected with 4 μg of wild type ABCB4, mutant ABCB4 or ABCC1 expression vector. Three days later, cells were fixed with methanol/acetone 4/1. The fixed cells were washed with PBS-0.05% Tween20 for 10 minutes and incubated with primary antibodies to ABCB4 (P3_II_26) or ABCC1 (R1)[26] in PBS, 0.05% Tween20, 10% fetal calf serum for 1 hour.

Glass slides were washed and incubated with goat anti-mouse IgG antibody Alexa fluor 594 conjugate (Molecular Probes) or goat anti-rat IgG antibody Texas-Red conjugate (Rockland) respectively. The cells were washed with PBS and embedded in mounting medium containing DAPI (Vector Laboratories). Pictures were taken using a fluorescence microscope (Leica) equipped with digital camera.

### Competition experiments with phosphatidylcholine vesicles and lentiviral supernatants

PC liposomes were generated by drying a 100 mg/ml L-α-phosphatidylcholine solution in chloroform (Sigma) under a nitrogen stream and resuspended by vortexing in 5 ml of Hank's Balanced Salt Solution (HBSS, Biowhittaker). A 500 μM solution was sonicated (amplitude 60, 50–60 Hz, Vibra Cell Sonicator, Sonics & Materials Inc) on ice for 10 minutes, filtered through a 0.45 μm filter and used immediately.

For competition experiments with viral supernatants generated in the presence of ABCB4 the target cells were incubated at saturating multiplicity of infection with CMVDsRed virus produced with wild type ABCB4 or mutant ABCB4. Cells were saturated with CMVDsRed/wild type ABCB4 by transduction at a multiplicity of infection of 5 which resulted in target cells that were 100% positive for DsRed. For the DsRed/mutant ABCB4 virus equal amounts of p24 were used.

At the same time as the competitors, PGKGFP vector was added at a multiplicity of infection of 0.5.

The PGKGFP transduction efficiency was determined after four days by flow cytometry.

### Phosphatidylcholine and cholesterol measurement

The amount of phosphatidylcholine in lentiviral vector pellets was determined by measuring choline by enzymatic assay with phospholipase D and choline oxidase[27]. Cholesterol was determined using homovanillic acid and cholesterol oxidase as decribed before[28]. Both enzymatic reactions were measured with a Novostar analyzer (BMG Labtech).

### High-performance thin-layer chromatography (TLC)

Membranes from 293T cells producing lentivirus were prepared by resuspension of cell pellets in 1 mM NaHCO3 and disruption of the cells using a potter homogenizer. Cellular debris was removed by centrifugation of 10 min at 4000 rpm in a Hettich tabletop centrifuge, membranes were harvested by ultracentrifugation for 2 hours at 20.000 rpm and resuspended in PBS containing 1% SDS.

Phospholipids were extracted from concentrated virus and cellular membranes, dissolved in chloroform/methanol (1:2) and run on silica gel 60 plates (Merck, Darmstadt, Germany) as described[29]. Spot densities were quantified via photodensitometric scanning using Quantity One-4.2.3 software (BioRad, Veenendaal, the Netherlands).

### Statistical analysis

Statistical analysis were performed using SPSS 10.0 software and significant differences were considered if P < 0.05 determined by One-Way ANOVA.

## Competing interests

The author(s) declare that they have no competing interests.

## Authors' contributions

NT, KH, RR, CP, MW, DM and JS designed and performed experiments. NT, RE and JS analysed data and wrote the manuscript.
